# Supplementary material for: Sucrosomial® Iron: An Updated Review of Its Clinical Efficacy for the Treatment of Iron Deficiency
Source: Pharmaceuticals (Basel). 2023 Jun 6;16(6):847. doi: 10.3390/ph16060847 (PMC10305423; doi:10.3390/ph16060847)
Supplement: Supplementary file 1 [file pharmaceuticals-16-00847-s001.zip › pharmaceuticals-2366168-supplementary.pdf]

Table S1. Sucrosomial iron (SI) administration in oncologic patients (11 studies, 294 patients)

Author (year)[Ref] Study type	Patients	Treatment Compound (Dose) Duration	Baseline Hb (g/dL)	Final Hb (g/dL)	Baseline Ferritin (ng/mL)	Final Ferritin (ng/mL	Baseline TSAT (%)	Final TSAT (%)	GI side effects
Renso et al (2015)[1] Case series	21 patients with lymphoproliferati-ve disease	SI (30 mg/day) +DEPO 150 mcg/week Chemotherapy 2 months	10.1	10.9	---	---	---	----	4%
Petrungaro et al. (2015)[2] Case series	10 patients with lymphoma (4 HL,6NHL)	SI (30 mg/day) After chemotherapy 2 months	10.0	11.2 (↑QoL)	43	93	---	---	?
Grillone et al. (2016)[3] Case series	30 patients with solid tumors	SI (30 mg/day) Chemotherapy 2 months*	11.2	11.3					10%
Romano et al. (2016)[4] Case series	25 Hodgkin lymphoma ≥2B	SI (30 mg/day) Chemotherapy End of treatment**	10.2	12.8	90	277	14.3	35.9	No
Sabbatini et al (2017)[5] Case series	30 patients with solid tumors	SI (30 mg/day) (n=15)<br>SI (60 mg/day) (n=15)<br>3 months**	10.5<br>9.8	12.0<br>12.0	---	---	---	---	Some dyspepsia and diarrhea
Poyato et al. (2017)[6] Case series	9 patients with solid tumors	SI (30 mg/day) Chemotherapy 2 months***	9.8	10.7	?	?	?	?	11%
Monari et al (2016)[7] Observational	15 Advanced prostate cancer with bone metastases	SI (30mg/day) (n=7)<br>No iron (n=8)<br>Chemoradiotherapy 6 months	11.1<br>10.9	12.2<br>9.7	---	---	---	---	?<br>Well tolerated
Barragans et al. (2016)[8] RCT pilot	15 patients with peritoneal carcinomatosis	SI (30mg/day) (n=8)<br>FS (80 mg/day) (n=7)<br>3 months	10.4<br>9.5	12.5<br>11.9	529<br>1048	---	10<br>8	---	25% mild<br>29% mild
Barzaghi et al. (2016)[9] Observational	15 patients advanced rectal cancer & bleeding	SI (30 mg/day) (n=11)<br>IV iron + folic acid (n=4)<br>21 days	8.0	11.4<br>11.6	100	---	25	---	No
Mafodda et al (2017)[10] RCT pilot	64 patients with solid tumor	SI (30 mg/day) +DEPO 500 mcg/3 w<br>FG (125 mg/wk)+ DEPO 500 mcg/3 weeks<br>2 months***	9.4<br>9.2	12.7<br>12.9	---	---	---	---	3%<br>0%
Zuccarini A et al (2022)[11] RCT	60 patients with chemotherapy-induced anemia	SI (30 mg/day) +ESA weekly for 12w<br>Control ESA weekly for 12w	9.5<br>9.2	52%#<br>31%	---	----	----	----	No

*2 patients started ESA; **No ESA or blood transfusion during study period, 70% reached delta Hb ≥2 g/dL or Hb ≥12 g/dL; *** one patient transfused. #% of patients attaining the haematological response, defined as a 2g/dL increase in Hb or Hb >12 g/dL. DEPO, darbepoetin; ESA, eyrthropoiesis stimulating agent; FG, ferric gluconate; FS, ferrous sulfate. RCT, randomized control trial.

References

1. Renso R, Bolis S, Casaroli I, et al. Use of darbopoietin and oral liposomal iron (Sideral Forte) in chemotherapy associated anemia in patients with lymphoproliferative disease. Expert Review of Hematology, 2015; 8 (Suppl 1):S24

2. Petrungaro A, Mineo G, Cingari R, et al. Benefits assessment of liposomal iron (Sideral Forte) administration to hematologic patients in follow-up after chemotherapy. Expert Review of Hematology, 2015; 8 (Suppl 1):S18-S19

3. Grillone F, Gualteri S, Ventura M, et al. Efficacy and tolerability of Sucrosomial® Iron in elderly (≥75 years) patients with solid tumors treated with anticancer agents: a retrospective analysis. Expert Review of Hematology, 2016; 9 (Suppl 1): 28.

4. Romano A, Conticello C, Motta G, et al. Oral Sucrosomial® Iron supplementation in patients affected by Hodgkin lymphoma with mild anemia before chemotherapy: an observational study. Expert Review of Hematology, 2016; 9 (Suppl 1): 30-31.

5. Sabbatini G, Bonfitto F. Effectiveness of two different dosage regimes of Sideral® forte in anemic patients with solid tumor. Expert Review of Hematology, 2017; 10 (Suppl 1): 24-25.

6. Poyato E, Villacé P, Pujades C, et al. Safety and efficacy of Sucrosomial® iron in cancer patients with anemia. Expert Review of Hematology, 2017; 10 (Suppl 1): 26-27.

7. Monari F, Morgantin AG, Frezza G, et al. Oral Sucrosomial® Iron (Sideral® Forte) supplementation in patients with advanced prostate cancer and bone metastasis treated with 223 radium dichloride. Expert Review of Hematology, 2016; 9 (Suppl 1): 24-25.

8. Barragans M, Camblor M, Cuerda C, et al. Sucrosomial® Iron versus ferrous sulfate for anemia in patients undergoing peritoneal carcinomatosis with cytoreductive surgery and hyperthermic intraperitoneal chemotherapy. Expert Review of Hematology, 2016; 9 (Suppl 1): 22-23.

9. Barzaghi D, Cristiano O, Rlmo M, Guida C. Sucrosomial® Iron and radiotherapy in the neoadjuvant treatment of rectal cancers. Good news for patients? Expert Review of Hematology, 2016; 9 (Suppl 1): 26-27.

10. Mafodda A, Giuffrida D, Prestifilippo A, Azzarello D, Giannicola R, et al. Oral Sucrosomial® iron versus intravenous iron in anemic cancer patients without iron deficiency receiving darbepoetin alfa: A pilot study. Support. Care Cancer. 2017; 25: 2779–2786.

11. Zuccarini A, Cicognini D, Tancredi R, Ferrari A, Rizzo G, Lasagna A, et al. Randomized trial of sucrosomial iron supplementation in patients with chemotherapy-related anemia treated with ESA. Support Care Cancer 2022; 30:7645-7653.

Table S2. Sucrosomial iron (SI) administration in patients with chronic kidney disease (CKD) (12 studies, 378 patients)

Author (year)[Ref] Study type	Patients	Treatment Compound (Dose) Duration	Baseline Hb (g/dL)	Final Hb (g/dL)	Baseline Ferritin (ng/mL)	Final Ferritin (ng/mL	Baseline TSAT (%)	Final TSAT (%)	GI side effects
Non-dialysis chronic kidney disease (ND-CKD)									
Pisani et al (2014)[1] RCT	99 ND-CKD3-5	SI (30 mg/day) (n=66)<br>FG (125 mg/week, TID:1000mg) (n=33)<br>3 months	10.8<br>10.7	11.4<br>11.7	71<br>68	86<br>239	16.5<br>17.0	18.3<br>21.5	12%<br>18%
Moussa-Abdi et al (2015)[2] Observational	28 ND-CKD	SI (60/mg/day) (n=14)*<br>FS (100 mg/day) (n=14)<br>3 months	11.2<br>11.3	11.7<br>11.4	78<br>182	90<br>228	17.9<br>22.3	22.4<br>31.8	14%<br>58%
Equitani et al. (2016)[3] Observational	16 ND-CKD Severe anemia	SI (60/mg/day)+ ESA (n=8)<br>No iron + ESA (n=8)<br>3 months	8.6<br>8.9	12.6<br>11.4	12<br>21	68<br>21	24<br>28	39<br>19	No
Cuzzola et al. (2016)[4] Case series	35 ND-CKD Intolerant to FS	SI (30 mg/day)<br>3 months	9.3	11.1	---	---	11.2	8.9	No
Dimikovic et al. (2018)[5] Case series	31 ND-CKD3-4	SI (30 mg/day)<br>ESA (no change in dosage)<br>6 months	10.2	10.3	213	169	26.8	24.4	Mild[###]
Arenas et al. (2016)[6] Case series	24 ND-CKD3-4	SI (30 mg/day)<br>6 months	11.1	12.8	34	75	13.8	26.1	No
Givreas et al. (2018)[7] Case series	40 ND-CKD3-5	SI (30 mg/day)<br>24 months	11.6	12	74	66	---	---	No
Montagud et al. (2020)[8] Case series	37 ND-CKD3 71% intolerant to conventional oral iron	SI (30 mg/day)<br>12 months	12.0	11.9	100	116	17.0	18.7	3%
Chronic kidney disease on hemodialysis (HD-CKD)									
Panichi et al. (2015)[9] RCT pilot	12 HD-CKD	SI (30-180 mg/week)<br>FG (30-180 mg/week)<br>3 months	12.7<br>12.0	12.7<br>12.6	---<br>---	---<br>---	24.0<br>27.6	21.0<br>30.8	No
Pistoni et al. (2016)[10] RCT pilot	22 HD-CKD	SI (360 mg/week)+ESA (n=13)**<br>FG (16-190 mg/week)+ESA (n=9)***<br>3 months	10.7<br>11.1	11.4<br>11.4	312<br>285	177<br>250	21.6<br>18.6	20<br>15.7	?

Manca et al. (2017)[11]	10 HD-CKD	Switched from FG (31.2 to 125 mg/week) to SI (30 mg/day)<br>15 months	12.2	11.8	1705	587	49.5	32.7	No
Reggiani et al. (2022)[12]<br>Case series	24 HD-CKD	Switched from FG (62.5 mg/week) to SI (90 mg/week)##<br>3 months	11.2	11.0	225	97	30	17	4%

FG, ferric gluconate; ESA, erythropoiesis stimulating agent; EPO, erythropoietin; RCT, randomized control trial; TSAT, transferrin saturation index; GI, gastro-intestinal.
*Need for EPO 36% with SI vs. 57% with FS.
**EPO dose decreased by 2000 IU/week in 38% and increased in 23% of SI patients.
***EPO dose increased by 2000 IU/week in 67% and decreased in 11% of FG patients.
##EPO dose increased by 2000 IU/week.
###More frequently mild dyspeptic symptom and less frequently constipation.

References:

1. Pisani A, Riccio E, Sabbatini M, Andreucci M, Del Rio A, et al. Effect of oral liposomal iron versus intravenous iron for treatment of iron deficiency anaemia in CKD patients: A randomized trial. Nephrol. Dial. Transplant. 2015; 30: 645–652.
2. Moussa-Abdi F, Alvaredo de Beas F, Velasco –Pilar R, et al. Hierro liposómico oral en ERCA. Experiencia con dos dosis diarias y comparación con sulfato ferroso (P.188). Nefrología 2015; 35 (suppl 1):54.
3. Equitani F. Erythropoietin (EPO) plus oral Sucrosomial® Iron versus EPO alone for the treatment of severe anemia in no end-stage chronic kidney disease. Expert Review of Hematology 2016; 9 (Suppl 1): 35-36.
4. Cuzzola C, Mancini A, Giancaspro V. Anemia in chronic kidney disease patients: comparison between Sucrosomial® iron and ferrous sulfate. Expert Review of Hematology 2016; 9 (Suppl 1): 33.
5. Dimkovic N, Maslarevic Radovic V, Jankovic A, et al. Is liposomal iron good alternative over IV iron for maintenance therapy in CKD patients [SP319]. Nephrology Dialysis Transplantation 2016; 31 (Suppl 1): i193–i199,
6. Arenas MD, Herrera AC, Chacón A, Alzate E. Efficacy, tolerance, and adherence to treatment with Sucrosomial® Iron in patients with chronic kidney disease stages 3–4 and iron deficiency. Expert Review of Hematology 2016; 9 (Suppl 1): 34-35.
7. Montagud-Marrahia E, Arrizabalaga P, Abellanab R, Pocha E. Liposomal iron in moderate chronic kidney disease. Nefrología 2020; 40: 446-452.
8. Griveas I. Efficacy and tolerability of oral Sucrosomial® iron in CKD patients with anemia. Expert Review of Hematology 2017; 10 (Suppl 1): 8-10.
9. Panichi V, Scatena A, Digiorgio A, Paoletti S. A randomized trial investigating the effects of oral liposomal iron (Sideral Forte) versus intravenous iron gluconate in CKD hemodialysis patients. Expert Review of Hematology 2015;8 (Suppl 1):19
10. Pistoni G, Di Martino M, Veziano E, Saffioti S. Effectiveness of Sucrosomial® Iron (Sideral® Forte) in dialysis patients in therapy with intravenous iron and erythropoietin (EPO). 57º Congresso Nazionale Societá Italiana di Nefrologia, Milano, October 2016.
11. Manca ML, S Mura S, Solinas R, Di Lauro L. Effects of substitution of intravenous iron therapy with oral sucrosomial® pyrophosphate iron in HD-CKD patients with hyperferritinemia – Results at 15 months. Expert Review of Hematology 2017: 10 S1
12. Reggiani F, Colombo G, Astori E, Landoni L, Finazzi S, Milzani A, et al. Preliminary experience on the use of sucrosomial iron in hemodialysis: focus on safety, hemoglobin maintenance and oxidative stress. Int Urol Nephrol 2022;54:1145-1153. doi: 10.1007/s11255-021-02983-8.

Table S3. Sucrosomial iron (SI) administration in patients with gastrointestinal disease (10 studies, 363 patients)

Author (year) [ref] Study type	Patients	Treatment Compound (Dose) Duration	Baseline Hb (g/dL)	Final Hb (g/dL)	Baseline Ferritin (ng/mL)	Final Ferritin (ng/mL	Baseline TSAT (%)	Final TSAT (%)	GI side effects
Inflammatory Bowel Disease									
Abbati et al. (2019) [1] Case series	30 IBD (17 CD, 13UC) Mild anemia	SI (30 mg/day) 3 months	11.7	12.3	46	45	11	17	13%
Bastida et al. (2021)[2] Case series	46 IBD (31CD; UC, 15CD, intolerant to FS)	SI (30 mg/day) 3 months	11.2	11.7 (↑QoL)	14	16	9	16	11%
Indriolo et al. (2014)[3] Observational	38 IBD Hb >9 g/dL	SI (30 mg/day) (n=13)<br>FS (105 mg/day) (n=14)<br>No iron (n=11)<br>3 months	10.6<br>10.9<br>11.4	12.6<br>12.3<br>11.9	---	---	---	---	No
Bertani et al. (2021)[4] RCT	42 UC Mild-to-moderate anemia	SI (60 mg/day, 2 months, plus 30 mg/day, 1 month)<br>FCM (1000 mg IV, at baseline)<br>3 months	11.1<br>10.3	12.2<br>11.8	16<br>10	26<br>131*	---	---	5%<br>0%
Stuklov et al. (2020)[5] Case-control	77 IBD** (23 CD, 44 UC)	SI (60 mg/day) (n=28)<br>IS (913.6 ± 269.6 mg cumulative) (n=22)<br>3 months	10.1<br>10.0	12.5<br>11.6	---	---	---	---	No
Celiac Disease									
Ragozzino et al (2015)[6] Case series	6 Celiac disease<br>28 Non-celiac gluten sensitivity	SI (30 mg/day 15d, 15 mg/day 75d) 3 months	8.8<br>9.7	11.5<br>12.5	13<br>18	23<br>29	---	---	---
Scorsone et al. (2016)[7] Observational	24 Celiac disease All T1DM	SI (30 mg/day) (n=12)<br>FS (105 mg/day) (n=12)<br>1 month	---<br>---	+1.27<br>+0.82	---	---	19.1<br>17.4	23.6<br>18.7	---
Elli L. et al. (2018)[8] Observational	43 Celiac disease	SI (30 mg/day) intolerant to FS (n=24)<br>FS (105 mg/day) (n=19)<br>3 months	10.9<br>11.0	12.0<br>12.9*	11<br>13	18<br>59*	10<br>11	15<br>20*	9%<br>23%
Bariatric surgery									
Ciudín et al. (2018)[9] Case-control	40 Bariatric surgery All women	SI (28 mg/day) (n=20)<br>IVI (Iron sucrose 300 mg) (n=20)<br>3 months	12.4<br>12.5	12.3<br>12.7	102<br>98	89<br>96	22.9<br>23.6	24.1<br>26.3	No
Greco et al. (2023)[10] Case series	17 Bariatric surgery	SI (60 mg/day) plus calcium, magnesium, zinc, water-soluble and fat-soluble vitamins<br>3 months	10.46	10.63	3,5	6.9			50%

CD, Crohn disease; FCM, ferric carboxymaltose; FS, ferrous sulfate; IS, Iron Sucrose; GI, gastro-intestinal; IBD, inflammatory bowel disease; IVI, intravenous iron; RCT, randomized controlled trial; T1DM, type 1 diabetes mellitus; TSAT, transferrin saturation index; UC, ulcerative colitis; (---) not reported. * $p<0.05$ SI vs. FS or FCM/FS; **27 patients was analyzed to determine anemia characteristics

References

1. Abbati, G.; Incerti, F.; Boarini, C.; Pileri, F.; Bocchi, D.; Ventura, P.; Buzzetti, E.; Pietrangelo, A. Safety and efficacy of sucrosomial iron in inflammatory bowel disease patients with iron deficiency anemia. Intern. Emerg. Med. 2018, 14, 423–431.
2. Bastida G, Herrera-de Guise C, Algaba A, Ber Nieto Y, Soares JM, Robles V, et al. Sucrosomial Iron Supplementation for the Treatment of Iron Deficiency Anemia in Inflammatory Bowel Disease Patients Refractory to Oral Iron Treatment. Nutrients 2021, 13, 1770. https://doi.org/10.3390/nu13061770.
3. Indriolo A, Ravelli P. Comparison between liposomial iron and ferrous sulfate in patients with iron deficiency anemia and inflammatory bowel disease. A pilot controlled study. J Crohn's Colitis 2014 Feb;8(Suppl 1):S289.
4. Bertani L, Tricò D, Zanzi F, et al. Oral Sucrosomial Iron is as effective as intravenous ferric carboxy-maltose in treating anemia in patients with ulcerative colitis. Nutrients 2021; 13: 608.
5. Stuklov NI, Basiladze IG, Kovalchuk MS, Pivnik AV, Knyazvev OV, Parfenov AI, Tarantino G. New options in management of iron-deficiency syndromes in inflammatory bowel diseases. EC Gastroenterology and Digestive System 7.8 (2020): 94-104.
6. Ragozzino g, Riccio A, Mattera E. Effectiveness of oral liposomal iron (Sideral¬ Forte) in patients with intestinal malabsorption (Celiac disease and gluten sensitivity). Exp Rev Hematol 2015; 8 (Suppl 1): 21-22.
7. Scorsone A, Calandrino C, Ferranti R, et al. Iron supplementation with liposomal formulation (Sideral¬ Forte) in celiac patients with iron deficiency and Type 1 diabetes mellitus. Exp Rev Hematol 2015; 8 (Suppl 1): 19-20.
8. Elli L, Ferretti F, Branchi F, Tomba C, Lombardo V, et al. Sucrosomial® iron supplementation in anemic patients with celiac disease not tolerating oral ferrous sulfate: A prospective study. Nutrients. 2018; 10: 330.
9. Ciudin A, Simó-Servat O, Balibrea JM, Vilallonga R, Hernandez C, Simó R, Mesa J. Response to oral sucrosomial iron supplementation in patients undergoing bariatric surgery. The BARI-FER study. Endocrinol. Diabetes Nutr. 2018, 65, 17–20.
10. Greco F, Pignata G, Marano L. Efficacy of food for special medical purposes (FSMPs) with Sucrosomial minerals (Sideralmed®) in the treatment of vitamins and mineral deficiencies developed after standard oral supplementation following hypoabsorptive bariatric surgery. Blood Transfus 2023; 21 (Suppl 1): S25-S26.

Table S4. Sucrosomial iron (SI) administration in cardiology patients (4 studies, 119 patients)

Author (year) [Ref] Study type	Patients	Treatment Compound (Dose) Duration	Baseline Hb (g/dL)	Final Hb (g/dL)	Baseline Ferritin (ng/mL)	Final Ferritin (ng/mL	Baseline TSAT (%)	Final TSAT (%)	GI side effects
Heart failure									
Marazia et al (2017)[1] Case series	9 patients with CHF (LVEF ≤39%)	SI (60 mg/day)* 1 month	10.3	11.0	32	67	---	----	?
Putorti et al. (2017)[4] Case series	10 patients with Hypertensive heart disease	SI (60 mg/day) 5 weeks	10.5	11.5	---	---	---	---	No
Karavidas et al. (2021)[3] Observational	50 patients with HFrEF (LVEF 27±5)	SI (28 mg/day), 3 months (n=25)** Matched non-treated controls (n=25) Follow-up 3 and 6 months	12.5 12.9	12.9 13.2 12.7 12.6	39 45	67 79 45 44	---	---	4% ---
Interventional cardiology									
Ruperto et al. (2017)[4] Observational	50 patients percutaneous coronary intervention	SI (30 mg/day) (n=25) FS (105 mg/day) (n=25) 3 months post-PCI	8.9 9.1	11.2 11.0	---	---	---	---	0% 32%

CHF, chronic heart failure; LVEF, left ventricular ejection fraction; HFrEF, heart failure with reduced ejection fraction; FS, ferrous sulphate; *BNP and CRP decreased with treatment; **B-natriuretic pepetide (BNP) and C-reactive protein (CRP) decreased with treatment, 6min walking distance (6MWD) increased after treatment, and NYHA (New York Heart Association) class improved.

References

1. Marazia S, Loderi S, Magliari F, et al. Sucrosomial® iron supplementation can be a useful support treatment in patients with heart failure and anemia. Exp Rev Hematol 2017; 10 (Suppl 1): 33.
2. Putorti G. Benefits of Sucrosomial® oral iron in patients with heart disease and concomitant iron-deficiency anemia. Exp Rev Hematol 2017; 10 (Suppl 1): 37.
3. Karavidas A , Troganis E, Lazaros G, Balta D, Karavidas I-N, Polyzogopoulou E, et al. Oral sucrosomial iron improves exercise capacity and quality of life in heart failure with reduced ejection fraction and iron deficiency: a non-randomized, open-label, proof-of-concept study. Eur J Heart Fail 2021;23(4):593-597. doi: 10.1002/ejhf.2092.
4. Ruperto C, Ricca G, Antonio AA, et al. Oral Sucrosomial® iron supplementation in patients underwent percutaneous coronary intervention: safety, efficacy and tolerability. Exp Rev Hematol 2017; 10 (Suppl 1): 33-34.

Table S5. Sucrosomial iron (SI) administration in Internal Medicine (10 studies, 468 patients)

Author<br>(year) [Ref]<br>Study type	Patients	Treatment<br>Compound (Dose)<br>Duration	Baseline<br>Hb<br>(g/dL)	Final<br>Hb<br>(g/dL)	Baseline<br>Ferritin<br>(ng/mL)	Final<br>Ferritin<br>(ng/mL	Baseline<br>TSAT<br>(%)	Final<br>TSAT<br>(%)	GI<br>side<br>effects
Nasuti et al.<br>(2016) [1]<br>Case series	30 IDA various origin	SI (60 mg/day)<br>2 months	9.8	12.0	---	---	---	---	10%
Campanella et al<br>(2015) [2]<br>Case series	16 IDA various origin	SI (30 mg/day) 40 days (n=8)<br>SI (30 mg/day) 60 days (n=8)	10.3<br>10.8	11.9<br>12.6	<20<br><20	----	----	---	No
Scifo et al<br>(2015) [3]<br>Case series	9 haemorrhoidal disease with IDA	SI (60 mg/day)<br>3 months	9.4	10.8	10	80	18	34	No
Giordano et al.<br>(2016) [4]<br>Observational	21 women with inflammatory anemia	SI (60 mg/day) (n=9)<br>FS (210 mg/day) (n=12)<br>3 months	8.5<br>9.0	11.5<br>9.5	100<br>120	260<br>100	<20<br><20	---	0%<br>42%
Parisi et al.<br>(2016) [5]<br>Observational	43 patients with systemic sclerosis	SI (60 mg/day) (n=21)*<br>FS (105 mg/day) (n=22)<br>3 months	10.2<br>10.7	13.4<br>11.9	130<br>110	240<br>150	---	---	0%<br>23%
Bellodi et al.<br>(2016) [6]<br>Observational	82 patients with IDA	SI (30 mg/day)<br>FG or FCM (500 mg) + SI (30 mg/day)<br>6-7 months	10.3<br>8.8	11.6<br>12.2	7.5<br>5	27.5<br>27	---	---	No<br>3.7%
Vallerio et al.<br>(2016) [7]<br>Case series	8 patientis with Hepatitis C-related cirrhosis	SI (60 mg/day)**	9.4	10.1	10	36	19	24	No
Marchitto et al.<br>(2018) [8]<br>Case series	43 elderly with IDA	SI (30 mg/day)<br>FG (62.5 mg)<br>1 month	10.5<br>8.9	11.9<br>9.9	---<br>---	---<br>---	---<br>---	---<br>---	---
Giordano et al.<br>(2019) [9]<br>RCT	135 patients with MSD and low-risk refractory anemia	SI (28mg/day) + EPO (n=54) (10.5 w)***<br>FG (62.5 mg/week)+ EPO (n=43) (12 w)***<br>No iron + EPO (n=38) (12 w)***<br>3 months	8.9<br>9.3<br>9.7	12.0<br>12.0<br>12.0	610.2<br>608.8<br>699.5	607.0#<br>723.7#<br>730.3#	---<br>---<br>---	32#<br>48#<br>40#	No
Giordano et al.<br>(2021) [10]<br>RCT	90 patient with IDA due to bleeding	SI (120 mg/day) (n=45)<br>FG (62.5 mg/day to cover TID) (n=45) (3.5 w)***<br>4 weeks $	8.5<br>8.2	12.0<br>12.0	5<br>7	260##<br>18##	---	---	36%<br>22%###

EPO, epoetin.alfa; FCM, ferric carboxymaltose; FG, ferric gluconate; IDA. iron deficiency anemia; MSD, myelodysplastic syndrome; TID, total iron deficit. * SI treatment reduced ESR and CRP levels; **Reduction of aortic stiffness;*** weeks median to achieve Hb 12 g/dL; #Ferritin/TSAT after 1 month; ## 12 months after enrollment; ###5 urticaria and headache and 5 hypotension; $ Cost 120 €/month for SI, 300 €/month for FG.

References

1. Nasuti A, Sagristani M, Sessa F. Oral sucrosomial® iron (Sideral® forte) is effective and well tolerated in elderly patients affected by iron deficiency anemia of various origin. Exp Rev Hematol, 2016; 9 (Suppl 1): S38-S39.
2. Campanella MP, Ebbli A, Gaibazzi D, et al. Effectiveness and compliance of oral liposomal iron (Sideral Forte) treatment for iron deficiency anemia: a valid alternative to iv iron therapies. Exp Rev Hematol 2015; 8 (Suppl 1): S31-S32.
3. Scifo M. Liposomal iron and ascorbic acid (Sideral Forte) supplementation in the treatment of iron deficiency anemia in patients with hemorrhoidal disease. Exp Rev Hematol 2015; 8 (Suppl 1): S21.
4. Giordano, G. Reduction of inflammatory markers with liposomal iron (Sideral®). Pre-clinical and clinical results. Exp. Rev. Hematol. 2016; 9 (Suppl. S1): S17.
5. Parisi S, Bruzzone M, Scarati M et al. Efficacy of Sucrosomial® Iron (Sideral® Forte) in the treatment of anemia in patients affected by systemic sclerosis. Exp Rev Hematol, 2016; 9 (Suppl 1): S21-S22.
6. Bellodi A, Molinari E, Genova C, et al. Retrospective evaluation of iron deficiency patients in a Northwestern Italy anemia ambulatory: experience with Sucrosomial® Iron. Exp Rev Hematol 2016; 9 (Suppl 1): S29.
7. Vallerio P, Stucchi M, Sirico D, Buono A, Rinaldi, Palazzina M, Giannattasio C. Sucrosomial® iron and aortic stiffness in cirrhotic patients. Exp Rev Hematol, 2016; 9 (Suppl 1): S42.
8. N. Marchitto, F. Sindona, M. Marcinnò, D. Marino, G. Z. Alexandru, F. Costanzo, S. G. Dalmaso, G. Raimondi. Role of administration of oral Sucrosomial® Iron and intravenous ferric gluconate in patients with secondary anaemia: effects on haemoglobin values and cardiovascular risk. Blood Transfus 2019; 17 (Suppl 1): S21.
9. Giordano G, Cutuli MA, Lucchesi A, Magnifico I, Venditti N, Vergalito F, Gasperi M, Di Marco R. Iron Support in Erythropoietin Treatment in Myelodysplastic Syndrome Patients Affected by Low-Risk Refractory Anaemia: Real-Life Evidence from an Italian Setting. Acta Haematol. 2020;143:155-162.
10. Giordano G, Napolitano M, Di Battista V, Luchessi D. Oral high-dose Sucrosomial® Iron vs. intravenous iron in sideropenic anemia intolerant/refractory to iron sulfate. Multicentric randomized study. Ann Hematol. 2021; 100: 2173-2176.

Table 6S. Sucrosomial iron (SI) administration in Patient Blood Management (9 studies, 1559 patients)

Author (year)[Ref] Study type	Patients	Treatment Compound (Dose) Duration	Baseline Hb (g/dL)	Final Hb (g/dL)	Baseline Ferritin (ng/mL)	Final Ferritin (ng/mL	ABT rate (%)	LOS (days)	GI side effects
Cardiac surgery									
Testa et al. (2017)[1] Case series	28 patients postoperative	SI (30 mg/day, months 1 and 3)[b] 3 months	10.0	12.4	334	63	---	---	No?
Grossi et al. (2017)[2] Case series	16 patients postoperative	SI (120 mg/day, 7 days from POD7; n=8) [c]<br>SI (60 mg/day, 14 days from POD7; n=8) [c]<br>1-2 week	9.7<br>10.0	10.4<br>11.0	---	---	---	---	No
Buioni et al. (2017)[3] Case series	22 patients postoperative	SI (120 mg/day)<br>3 weeks	10.0	12.0	---	---	---	0	No
Pierelli et al. (2021)[4] RCT	1000 patients preoperative	SI (60 mg/day, 30 days; n=500)<br>Control (standard preparation; n=500)	---<br>---	13.9<br>13.3	---<br>---	184<br>160	65[a]<br>35	13<br>15	1.8%<br>---
Pagliani et al. (2017)[5] Observational	16 patients postoperative	SI (60 -120 mg/day) (n=8)<br>FS (105 mg/day) (n=8)<br>14 days	9.5<br>9.2	10.1<br>9.9	500<br>600	400<br>870	---	---	0%<br>33%
Venturini et al. (2022)[6] Observational	106 patients postoperative	SI (120 mg/day, 10 days + 30 mg/day, 10 days; n=54)<br>FCM (1000 mg IV, single dose; n=52)	10.1<br>10.1	12.0<br>12.5	411<br>386	220<br>689	---<br>---	---<br>---	No<br>No
Vascular surgery									
Lucertini et al. (2020)[7] RCT	51 patients AAAR Postoperative	SI (30 mg/day, 30 days; starting PO10; n=26)<br>Control (no iron; n=25)	9.3<br>9.3	11.2<br>9.7	---	---	0<br>0	---	No
Orthopedic surgery									
Di Costanzo (2017)[8] Observational	20 THR preoperative	SI (30 mg/day, for 30 days; n=10)<br>Control (No iron; n=10)	10.5<br>12	11.5[e]<br>12			0<br>40		
Scardino et al. (2019)[9] Observational	100 THR ID[e]<br>100 THR ID<br>100 THR non-ID preoperative	No iron<br>SI (30 mg/day, for 3-4 weeks)<br>No iron<br>All received tranexamic acid (1 g)	13.5<br>13.5<br>14.8	10.2[f]<br>13.3<br>12.8	66<br>65<br>160	---	7<br>0<br>0	6.5<br>4<br>4	---<br>No<br>---

AAAR, abdominal aortic aneurysm repair; FCM, ferric carboxymaltose; FS, ferrous sulphate; ID, iron deficiency; RCT, radommized control trial; THR, total hip replacement. [a] Compared to control, SI treatment resulted in reduced transfusion rate (35% vs. 65%, respectively; p<0.001) and transfusion index (0.95 units/pt vs. 2.03 units/pt, respectively; p>0.001). [b]6MWD increased and BNP decreased after treatment. [c] 6MWD increased after treatment. [d]ID defined by ferritin < 100 ng/mL, or ferritin levels > 100 ng/mL but C-reactive protein > 3 mg/L and transferrin saturation (TSAT) <20%. [e]Postop Hb 8.5 g/dL and 7.5 g/dL, for SI and control, respectively. [f]Final Hb was measured at POD30; SI administration resulted in cost saving 1763 €/pt, ID-SI vs. ID-no iron

References

1. Testa R, Venturini E, Sansoni C, et al. Oral Sucrosomial® Iron in post cardiac surgery patients. Efficacy and tolerability in a follow up of 3 months. Exp Rev Hematol 2017; 10 (Suppl 1): 12-13.
2. Grossi A, Baldari F, Codraro S, et al. Sucrosomial® iron: short-term efficacy compared to administration of 4 capsules/day for 7 days vs. 2 capsules/day for 14 days in post cardiac surgery patients for myocardial revascularization. Exp Rev Hematol 2017; 10 (Suppl 1): 31.
3. Buioni D, Nardella S, Maselli D. Effect of Sucrosomial® iron in early time after cardiac surgery. Exp Rev Hematol 2017; 10 (Suppl 1): 36-37.
4. Pierelli L, De Rosa A, Falco M, et al. preoperative Sucrosomial iron supplementation increases haemoglobin and reduces transfusion requirements in elective heart surgery patients: A prospective randomized study. Surg Technol Int 2021; 39:321-8.
5. Pagliani L, Payadattil S, Marigo L, et al. Hospital protocol for evaluating effectiveness and timing of use of Sucrosomial® iron in cardiac rehabilitation departments. Exp Rev Hematol 2017; 10 (Suppl 1): 35-36.
6. Venturini E, Masini G, Giallauria F. Short-term treatment of iron deficiency anemia after cardiac surgery. Int J Cardiol Heart Vasc 2022; 40:101038.
7. Lucertini G, Gazzola V, Boschetti GA, Khourieh T. Sucrosomial iron in the postoperative period after abdominal aortic aneurism repair. Blood Transfus 2020; 18 (Suppl 1): s25.
8. Di costanzo. Effectiveness of oral Sucrosomial iron (Sideral Forte) administration in patients undergoing hip replacement. Exp Rev Hematol 2017; 10 (Suppl 1): 15-16.
9. Scardino M, Di Matteo B, Martorelli F, Tanzi D, Kon E, D'Amato T. Improved patient blood management and cost saving in hip replacement surgery through the implementation of pre-operative Sucrosomial® iron supplementation: a quality improvement assessment study. Inter Orthop 2019; 43:39-46.
